# Maternal Bean Consumption during Pregnancy: Distribution and Nutritional Outcomes

**DOI:** 10.3390/nu15092234

**Published:** 2023-05-08

**Authors:** Bokun Yang, Mariyam S. Ferdousi, Julianna Morris, Rose H. Durnell, Daren Chan, Neila Rekić, Todd C. Rideout, Xiaozhong Wen

**Affiliations:** 1 Department of Exercise and Nutrition Sciences, School of Public Health and Health Professions, State University of New York at Buffalo, Buffalo, NY 14214, USA; bokunyan@buffalo.edu (B.Y.); jmorris7@buffalo.edu (J.M.); rideout@buffalo.edu (T.C.R.); 2 Division of Behavioral Medicine, Department of Pediatrics, Jacobs School of Medicine and Biomedical Sciences, State University of New York at Buffalo, Buffalo, NY 14214, USA; msferdou@buffalo.edu (M.S.F.); rosedurn@buffalo.edu (R.H.D.); darencha@buffalo.edu (D.C.); neilarek@buffalo.edu (N.R.); 3 Department of Epidemiology and Environmental Health, School of Public Health and Health Professions, State University of New York at Buffalo, Buffalo, NY 14214, USA

**Keywords:** maternal nutrition, pregnancy nutrition, HEI score, dried bean, chili, bean soup, dietary pulses, bean consumption, socioeconomic status, Hispanic population

## Abstract

(1) Background: Due to their high nutritional value, we aimed to characterize the frequency and amount of maternal consumption of beans during pregnancy and their associations with diet quality and nutrient intake. (2) Methods: We conducted a secondary data analysis of US pregnant women (*n* = 1444) from the Infant Feeding Practices Study II, a longitudinal study that followed mother–infant pairs from late pregnancy to 1 year postpartum. Maternal bean intake (food types [dried beans, chili, and bean soup], frequency, serving size, and amount), diet quality (Healthy Eating Index [HEI]), and nutrient intake were estimated with a Food Frequency Questionnaire taken in the third trimester of pregnancy. Associations of bean consumption with diet quality and nutrient intake were examined with analysis of variance, Fisher’s least significant difference tests, correlation coefficients, and coefficients of determination. (3) Results: In general, maternal bean consumption was low during pregnancy: 0.31 cups/week of dried beans, 0.37 cups/week of chili, and 0.10 cups/week of bean soup. Maternal bean consumption varied by socio-demographics and geographic regions. In comparison with those who never consumed dried beans, mothers who ate dried beans ≥ 1 time per week had a higher mean HEI score (67.5 vs. 63.6), intake of total fiber (24.4 vs. 17.4 g/day), and protein (93.4 vs. 79.9 g/day), but a lower percentage of energy from added sugar (12.6 vs. 15.2%). Higher dried bean consumption had weak-to-moderate correlations with intake of total fiber (correlation coefficient, 0.320), insoluble fiber (0.316), soluble fiber (0.310), and folate (0.286). Similar but less extensive correlations were observed for chili and bean soup consumption. (4) Conclusions: In this US cohort of pregnant women, bean consumption was low. Increased intake of beans (≥1 time per week) may improve maternal diet quality during pregnancy.

## 1. Introduction

Nutrition during pregnancy and the postpartum period is essential to ensure optimal health of both the mother and child. However, dietary nutrient intake amongst women of reproductive age in the US does not meet current dietary recommendations [1]. Previous work suggests that maternal pregnancy intake of energy, sugar, and saturated fat exceeds recommendations, whereas micronutrients and fiber are underconsumed [1]. Increased consumption of dietary pulses, specifically beans, could be a means to improve nutrient intake during pregnancy [2] as they are high in protein, fiber, complex carbohydrates, folate, zinc, iron, and magnesium with low saturated and total fat [3].

In non-pregnant populations, regular bean consumption has been associated with a range of positive health benefits including increased satiety [4], maintenance of a healthy body weight [5], improved glycemic control [6], and decreased blood lipids [7]. Although studies examining the benefits of bean consumption during pregnancy have been inadequate, there is research suggesting that increased maternal bean consumption may influence birth outcomes. For example, a previous study in pregnant Spanish women found an inverse association between dietary pulse consumption and the risk of small-for-gestational-age birth [8].

Although the recommended intake of dietary pulses for adults is 1.5 cups per week [9], consumption amongst the general population is below current recommendations, estimated at only 0.15 cups/day [4]. This low rate of bean consumption may be due to multiple factors including digestibility issues, access to beans, cultural practices, lack of preparation time, insufficient knowledge about bean nutrition, and taste preferences [4]. However, we are not aware of any previous reports that have specifically examined bean consumption patterns during pregnancy, which is a substantial research gap. Therefore, the objectives of this study were to compare consumption of beans between pregnant and non-pregnant women, identify socio-demographic correlates for bean consumption during pregnancy, and examine the correlations of bean consumption during pregnancy with maternal diet quality and nutritional outcomes.

## 2. Material and Methods

### 2.1. Participants and Setting

We analyzed secondary data from the Infant Feeding Practices Study II (IFPS II), a US longitudinal birth cohort that tracked mother–child dyads from pregnancy to 1 year postpartum [10]. IFPS II was conducted by the US Food and Drug Administration (FDA) and the Centers for Disease Control and Prevention (CDC) from 2005 to 2007. We included 2961 IFPS II participants, of which 1444 were pregnant women (prenatal group) and 1517 were non-pregnant/non-postpartum women (control group). The control included non-pregnant women who were aged 18 to 40 years, had not given birth in the previous 12 months, and had not taken part in the IFPS II study before. The prenatal group included pregnant women who completed the Dietary History Questionnaire (DHQ) before the infant delivery. When the DHQ was received from participants, some were disqualified, returned, and deleted due to extreme calorie report. In the prenatal group, 1756 DHQs were mailed and 1444 were qualified. In the control group, 2070 DHQs were mailed and 1517 were qualified. The FDA’s Research Involving Human Subjects Committee and the US Office of Management and Budget approved all study procedures. Mothers signed consent forms to participate in the IFPS II. This secondary data analysis was approved by the University at Buffalo Institute Review Board.

### 2.2. Exposure Measures

*Dried bean.* In the late pregnancy (3rd trimester) survey in IFPS II, prospective mothers reported the frequency of their dried bean intake in the past month as a part of the Food Frequency Questionnaire (FFQ). The question was “*Over the past month, how often did you eat cooked dried beans (such as baked beans, pintos, kidney, blackeyed peas, lima, lentils, soybeans, or refried beans)? (Please don’t include bean soups or chili.)*” The original answer options were never, 1 time per month, 2–3 times per month, 1 time per week, 2 times per week, 3–4 times per week, 5–6 times per week, 1 time per day, and 2 or more times per day. To preserve statistical power for further analysis, we combined the less common options and re-categorized answers into never, 1 time per month, 2–3 times per month, and 1 or more times per week. If mothers consumed dried beans, they further reported the servings usually consumed. The original answer options were less than ½ cup, ½ to 1 cup, and more than 1 cup. We calculated the dried bean amount (cup/week) based on the frequency and serving size. To facilitate calculation, we mathematically converted the frequency options to 0, 0.230, 0.575, 1, 2, 3.5, 5.5, 7, and 14 times per week. Serving options were converted to 0 (never), 0.25 (the midpoint of “less than ½ cup”), 0.75 (the midpoint of “½ to 1 cup”), and 1 cup (the lower bound of “more than 1 cup”). As established by the Dietary Guidelines for Americans (DGA) 2020–2025 [9], we defined meeting the national recommendation as the consumed dried bean amount being 1.5 or more cups per week.

*Chili.* Similarly, participants were asked *“How often did you eat chili?”.* The original answer options of never, 1 time per month, 2–3 times per month, 1 time per week, 2 times per week, 3–4 times per week, 5–6 times per week, 1 time per day, and 2 or more times per day. Similar to dried beans, we re-categorized these answers to never, 1 time per month, 2–3 times per month, and 1 or more times per week. If the mothers consumed chili, they were further asked *“Each time you ate chili, how much did you usually eat?”*. The answer options included less than ½ cup, ½ to 1 ¾ cups, and more than 1 ¾ cups. We calculated the chili amount (cup/week) based on the frequency and serving size. To facilitate calculation, we mathematically converted the frequency options to 0, 0.230, 0.575, 1, 2, 3.5, 5.5, 7, and 14 times per week. Serving options were converted to 0 (never), 0.25 (the midpoint of “less than ½ cup”), 1.125 (the midpoint of “½ to 1 ¾ cups”), and 1.75 (the lower bound of “more than 1 ¾ cups”).

*Bean soup.* For bean soup, participants were first asked, “*Over the past month, how often did you eat soups?*” The original answer options were never, 1 time per month, 2–3 times per month, 1 time per week, 2 times per week, 3–4 times per week, 5–6 times per week, 1 time per day, and 2 or more times per day. We summarized them to never, 1 time per month, and 2 or more times per month. If mothers consumed soups, they were then asked, “*Each time you ate soup, how much did you usually eat?*”. The answer options were less than 1 cup, 1 to 2 cups, and more than 2 cups. Then, the participants were asked, “*How often were the soups you ate bean soups?*” The original answer options were almost never or never, about ¼ of the time, about ½ of the times, about ¾ of the time, and almost always or always. We calculated the bean soup amount (cup/week) by multiplying the soup consumption frequency (0, 0.230, 0.575, 1, 2, 3.5, 5.5, 7, and 14 times per week), serving size (0, 0.5, 1.5, and 2 cups) by the proportion of soup being bean soup (0%, 25%, 50%, 75%, and 100%).

### 2.3. Nutritional Outcome Measures

In IFPS II, Diet*Calc was used to analyze the DHQ data and estimate the amount of nutrients obtained from the participants’ diet. Diet*Calc is software from the National Cancer Institute (NCI) to analyze nutrient content and food intakes based on the US Department of Agriculture’s (USDA) Food and Nutrient Database for Dietary Studies (FNDDS) and USDA’s MyPyramid Equivalents Database [11]. In this study, we focused on two categories of nutrients: (1) nutrients rich in bean foods (e.g., fiber), and (2) nutrients that potentially decrease with increased consumption of bean foods (e.g., added sugar). They included total energy intake (kcal/day), total carbohydrate (g/day), percentage of energy from carbohydrate (%), added sugar (tsp/day or g/day), percentage of energy from added sugar (%), total fiber (g/day), insoluble fiber (g/day), soluble fiber (g/day), protein (g/day), percentage of energy from protein (%), total fat (g/day), percentage of energy from total fat (%), monounsaturated fat (g/day), percentage of energy from monounsaturated fat (%), polyunsaturated fat (g/day), percentage of energy from polyunsaturated fat (%), saturated fat (g/day), percentage of energy from saturated fat (%), iron (mg/day), potassium (mg/day), folate (mcg/day), vitamin D (mcg/day), magnesium (mg/day), and total HEI-2005 score. We adjusted nutrient intakes of total fiber (g/1000 kcal), insoluble fiber (g/1000 kcal), soluble fiber (g/1000 kcal), iron (mg/1000 kcal), folate (mcg/1000 kcal), vitamin D (mcg/1000 kcal), and magnesium (mg/1000 kcal) by the total energy intake. We calculated maternal alternate Healthy Eating Index-2005 (HEI-2005) during pregnancy [12], based on DGA recommendations [13,14]. HEI-2005 includes 9 adequacy components which consist of total fruit, whole fruit, total vegetables, dark green and orange vegetables and legumes, total grains, whole grains, milk, meat and beans, and oils. The HEI-2005 also includes 3 moderation components which consist of saturated fat, sodium, and calories from solid fats, alcoholic drinks, and added sugars. HEI-2005 has a maximum total score of 100 and a higher score represents higher dietary quality.

### 2.4. Correlates

Based on the literature [15,16], we accounted for the following socio-demographic and behavioral measures as correlates of bean consumption during pregnancy: race/ethnicity (non-Hispanic White, non-Hispanic Black, Hispanic, and Asian/Pacific Islander/other), highest education level (1–8 years grade school, high school, 1–3 years college, college graduate, and postgraduate), employment status (unemployed and employed), household size (1–2, 3, 4, and ≥5 people), annual household income (<USD 25,000, USD 25,000 to <USD 40,000, USD 40,000 to <USD 60,000, and ≥USD 60,000), Special Supplemental Nutrition Program for Women, Infants, and Children [6] recipient status (non-recipient and recipient), regions of residency (New England, Middle Atlantic, East North Central, West North Central, South Atlantic, East South Central, West South Central, Mountain, and Pacific), smoking status during pregnancy (yes and no), and vegetarian diet status during pregnancy (yes and no). The information on these characteristics was self-reported by participants using a survey questionnaire at enrollment.

### 2.5. Statistical Analysis

In the descriptive analysis on socio-demographic characteristics, we used the frequencies and percentages for the categorical variables (e.g., race/ethnicity) and mean ± standard deviation [4] for the continuous variables (e.g., age) (Table 1). Additionally, in the total sample, we stratified the sample by pregnancy status and compared these socio-demographic measures between pregnant and non-pregnant/non-postpartum women by the chi-square test (for categorical variables) and *t*-test (for continuous variables). To examine the potential differences in bean consumption (dried beans, chili, and bean soup) between pregnant and non-pregnant/non-postpartum women, we used chi-square tests for the consumption frequency, serving size, and meeting national recommendations, as well as analysis of variance (ANOVA) for the consumption amount (cup/week) (Table 2). To identify the correlates of bean consumption, we focused on the differences in the consumption amount and performed ANOVA tests for overall differences and Fisher’s least significant difference (LSD) tests for pairwise comparisons between the categories of a specific correlate (Table 3). We performed ANOVA tests and LSD tests to examine the associations between bean consumption frequency and nutritional outcomes (Table 4). For dried bean and chili consumption, we used 4 frequencies (never, 1 time per month, 2–3 times per month, and 1 or more times per week). We condensed it to 3 frequencies (never, 1 time per month, and 2 or more times per month) for bean soup due to its lower consumption to ensure a sufficient sample size. In addition, we calculated the Pearson correlation coefficients (r) and coefficients of determination (r^2^) to assess the correlations of the bean consumption amount (cup/week) with nutritional outcomes (Table 5).

## 3. Results

### 3.1. Sample Characteristics

Table 1 shows the socio-demographic characteristics of the analytical sample (*n* = 2961), including 1444 pregnant and 1517 non-pregnant/non-postpartum women. In the total sample, 85.5% were White and 3.4% were Black. The average age was 28.8 years, 64.3% were employed, 59.0% had a household income of ≥USD 40,000/year, and 4.1% were vegetarian. Pregnant women were less likely to be White (83.5% vs. 88.3%) and fewer (55.1% vs. 62.7%) were likely to have a household income of ≥USD 40,000/year compared to the non-pregnant/not-postpartum women.

### 3.2. Distribution of Dried Bean Consumption and Differences by Pregnancy Status

Table 2 demonstrates a comparative analysis between pregnant and non-pregnant/non-postpartum women for the distribution of bean consumption in the form of dried beans, chili, and bean soup. Specifically, 55.0% pregnant and 54.2% non-pregnant/non-postpartum women consumed dried beans; 35.3% pregnant women and 39.8% non-pregnant/non-postpartum women consumed chili; 18.2% of pregnant and 19.4% of non-pregnant/non-postpartum women consumed bean soup. Only 6.7% and 7.0% of pregnant and non-pregnant/non-postpartum women, respectively, met the current recommendation of dried bean consumption (≥1.5 cups/week). On average, pregnant women consumed only 0.31 cups/week of dried beans, 0.37 cups/week of chili, and 0.10 cups/week of bean soup. Similarly, non-pregnant/non-postpartum women consumed 0.32 cups/week of dried beans, 0.44 cups/week of chili, and 0.11 cups/week of bean soup.

### 3.3. Correlates of Bean Consumption

Table 3 demonstrates the distribution of the frequency of maternal bean consumption by the socio-demographic and behavioral characteristics. Maternal dried bean consumption varied by race/ethnicity (*p* < 0.001) and geographic residency regions (*p* < 0.001). Hispanic mothers on average consumed 0.64 cups/week dried beans. Chili consumption was significantly higher among Black mothers with an average of 0.33 cups/week (*p* < 0.001). Living in East (0.43 cups/week) or West (0.42 cups/week) South Central regions was associated with relatively high dried bean consumption. There was no significant difference in dried bean and bean soup consumption by education level, employment status, household size, household income, or smoking status during pregnancy. However, mothers without a college degree reported consuming more chili (0.18 cups/week) than mothers with a college degree (0.14 cups/week). Mothers living in a household with three people had the lowest chili consumption (0.11 cups/week), compared to other mothers. Those living in the New England or Middle Atlantic region consumed the least (0.10 cups/week) amount of chili, while those living in the East South Central region consumed the most amount of chili (0.26 cups/week). Vegetarian mothers consumed more dried beans (0.61 cups/week, *p* < 0.001) and bean soup (0.30 cups/week, *p* < 0.001) compared to non-vegetarian mothers (0.30 cups/week and 0.09 cups/week, respectively). There was no association between the vegetarian diet and chili consumption (*p* = 0.230).

### 3.4. Associations between Frequency of Bean Consumption and Nutritional Outcomes

Table 4 displays a detailed breakdown of nutritional outcomes associated with the frequency of consuming dried beans, chili, or bean soup. The associations with nutritional outcomes were overall similar across the three bean foods. Compared to mothers who did not consume dried beans (group a in the Table 4), those who consumed them one or more times a week (group d) had a significantly higher intake of total energy, total fiber, insoluble fiber, soluble fiber, protein, total fat, monounsaturated fat, polyunsaturated fat, iron, potassium, folate, and magnesium, as well as a higher HEI-2005 total score. 

Mothers who consumed dried beans one or more times a week (group d) had the highest total food energy (2384.8 ± 945.4 kcal/day) and mothers who consumed dried beans one time a month (group b) had the lowest total food energy (2053.3 ± 829.1 kcal/day). However, the difference between the total food energy among the frequency groups was not vast. Similarly, mothers who consumed bean soup two or more times a month (group c) had the highest total food energy (2508.3 ± 1020.7 kcal/day) and mothers who never consumed bean soup (group a) had the lowest total food energy (2123.3 ± 910.2 kcal/day). The difference between total food energy (kcal/day) was greater among chili consumers. Mothers who consumed chili one or more times a week (group d) had the highest total food energy (2899.5 ± 1322.7 kcal/day) and mothers who never consumed chili (group a) had the lowest total food energy (2071.3 ± 874.9 kcal/day). Mothers who consumed bean soup two or more times a month (group c) had similar nutritional outcomes as those who consumed dried beans one or more times a week (group d) except for lower percentage of energy from saturated fat. Mothers who consumed dried beans one or more times a week (group d) had the highest energy-adjusted total fiber intake (10.5 ± 2.8 g/1000 kcal) and mothers who never consumed dried beans (group a) had the lowest total fiber intake (8.4 ± 2.6 g/1000 kcal). Additionally, mothers who consumed dried beans one or more times per week (group d) had the highest energy-adjusted insoluble (7.0 ± 1.9 g/1000 kcal) and soluble (3.5 ± 0.9 g/1000 kcal) fiber intake, and mothers who never consumed dried beans (group a) had the lowest energy-adjusted insoluble and soluble fiber intake (5.5 ± 1.9 and 2.8 ± 0.8 g/1000 kcal, respectively). Similar trends were found among two-or-more-times-a-month bean soup consumers (group c) with the highest energy-adjusted total fiber (11.5 ± 3.3 g/1000 kcal), insoluble fiber (7.6 ± 2.3 g/1000 kcal), and soluble fiber (3.8 ± 1.0 g/1000 kcal). Mothers who never consume bean soup (group a) had the lowest intake of energy-adjusted total fiber (8.7 ± 2.7 g/1000 kcal), insoluble fiber (5.7 ± 1.9 g/1000 kcal), and soluble fiber (2.9 ± 0.8 g/1000 kcal). In contrast, mothers who consumed dried beans one or more times a week (group d) and bean soup two or more times a month (group c) had a lower percent energy intake from added sugar. Unlike dried beans and bean soup, those who reported consuming chili one or more times a week (group d) did not have significant differences in the percentage of energy from added sugar, compared with never consumers (group a) (*p* = 0.115). 

Among dried bean consumers, group a had the highest energy-adjusted vitamin D intake (3.6 ± 2.1 mcg/1000 kcal) and group d had the lowest energy-adjusted vitamin D intake (3.1 ± 1.5 mcg/1000 kcal) (*p* < 0.001). Energy-adjusted magnesium intake was higher in group d (169.3 ± 31.6 mg/1000 kcal) and lower in group a (157.4 ± 34.1 mg/1000 kcal) (*p* < 0.001). Among chili consumers, group d had the highest energy-adjusted folate intake (141.0 ± 38.2 mcg/1000 kcal, *p* = 0.015). Among bean soup consumers, group c had the highest energy-adjusted iron (9.0 ± 1.9 mg/1000 kcal), potassium (1794.7 ± 287.0 mg/1000 kcal), folate (157.3 ± 51.9 mcg/1000 kcal), and magnesium (179.7 ± 33.4 mg/1000 kcal) (*p* < 0.001) intake. 

### 3.5. Correlation of Bean Consumption with Nutritional Outcomes

Table 5 reports the correlations (r) between bean consumption and nutritional outcomes, as well as the corresponding coefficients of determination (r^2^). Higher consumption of dried beans had weak-to-moderate correlations with total fiber (r = 0.320), insoluble fiber (0.316), soluble fiber (0.310), and folate (0.286). Bean soup and chili consumptions had relatively weak correlations with these nutritional outcomes. Corresponding correlations with the total HEI scores were relatively weak: dried beans (0.117), chili (0.030), and bean soup (0.130). Furthermore, the magnitude of the correlations of dried bean or chili consumption with most of the other nutritional outcomes were higher than that of bean soup consumption.

### 3.6. Supplemental Analysis on Bean Consumption and BMI

Supplemental Table S1 reports the association between bean consumption frequency and BMI. Dried bean (*p* = 0.430) or chili (*p* = 0.773) consumption frequency and BMI did not have a statistically significant association. However, overall, mothers who never consumed bean soup had the highest mean BMI (26.7 ± 6.9 kg/m^2^); the mean BMI was 25.4 ± 6.6 kg/m^2^ among mothers who consumed bean soup one time a month, and mothers who consumed bean soup two or more times a month had the lowest mean BMI (24.8 ± 5.8 kg/m^2^, *p* = 0.015). The BMI categories also differed significantly by bean soup consumption (*p* = 0.017): among never consumers (group a), 49.6% were underweight/normal, 23.9% were overweight, and 26.5% were obese; among one-time-a-month consumers (group b), 59.8% were underweight/normal, 21.5% were overweight, and 18.7% were obese; among two-or-more-times-a-month consumers (group c), 65.9% were underweight/normal, 19.5% were overweight, and 14.6% were obese. 

## 4. Discussion

From this US national pre-birth cohort, we characterized maternal bean consumption (dried beans, chili, and bean soup) during pregnancy, identified its significant correlates, and examined its correlations with maternal diet quality and nutritional outcomes. We found that overall, maternal bean consumption was low. Chili was more frequently consumed among less-educated participants. The amount of dried beans consumed varied greatly by race/ethnicity and geographic region. Pregnant women who ate more than 1 cup of dried beans per day had a higher intake of protein, dietary fiber, folate and several other micronutrients, and a higher HEI score, but a lower intake of added sugar (% energy), compared to non-consumers. Similar but less extensive associations were observed for chili and bean soup consumption.

We are unaware of any other studies that have focused on bean consumption among only pregnant and non-pregnant/non-postpartum women rather than the general population. We found that that both groups of pregnant and non-pregnant/non-postpartum women consumed below the recommended amount of beans (1.5 cups/week). We found that, on average, pregnant women consumed 0.31 cups/week, and non-pregnant/non-postpartum women consumed an average of 0.32 cups/week of dried beans. This finding is similar to other studies that have recorded dietary bean consumption rates in the general population. From an analysis of National Health and Nutrition Examination Survey (NHANES) data from 1999–2002, Mitchell et al. reported that only 7.9% of Americans consumed pulses on a given day [3]. A more recent updated analysis of NHANES respondents from 2003 to 2014 found no significant change over time in per capita pulse intake and reported an average intake of 71 grams of pulses per week, which is below the recommended 246–300 grams per week [15].

We observed a consistent trend in bean consumption among those from the low education levels (grades 1–8 and high school) through the high education levels (1–3 years of college, college education, and postgraduate education). The low education levels consumed less than half the amount of dried beans daily compared to the high education levels. This observation is supported by previous work reporting that beans were more likely to be consumed in college-educated adults compared with high-school-or-lower-educated individuals [15]. It is likely that a higher educational status would improve access to nutritional knowledge about the positive health attributes of beans and lead to higher consumption levels. In contrast to the pattern observed with dried beans, chili consumption was higher among the lowest education levels (grades 1–8). Those with a lower education level may consume more chili as convenient pre-prepared canned options or home-made chili prepared with low-cost ingredients. In a previous study that investigated how socioeconomic inequalities influenced eating behaviors, a lack of time due to work commitments was perceived as a barrier to preparing healthy foods amongst low and middle socioeconomic status (SES) women [17]. Low SES women were also less likely to try new recipes or experiment with nutritional changes, a factor which might also have contributed to the higher intake of chili we observed amongst low SES participants, as chili may be viewed as a traditional comfort food in the US. Further, Turrell and Kavanagh [18] reported that lower- vs. higher-educated individuals were more likely to buy high fat foods, such as chili [18]. Taken together, these data may indicate that chili may be consumed more for the sake of convenience, rather than for its health benefits, as it is often made with high fat beef and eaten with cheese and sour cream. 

The rate of maternal bean consumption in this study varied greatly by race/ethnicity. The highest consumption of dried beans and bean soup was among participants with Hispanic origin. In support of our findings, a previous NHANES study from 2003–2014 reported that Mexican-Americans and Hispanics were more likely to consume pulses or dried beans than individuals of other ethnicities [15]. The high rate of bean consumption among Hispanics correlates to the typical diet consumed in Latin America. According to the USDA food consumption survey, people of Hispanic origin consume the most cooked dried beans of any ethnicity [16]. In addition, our data suggest that women living in South Central regions consume more dried beans than other regions in the US. This observation is similar to a previous report demonstrating that the majority of dried beans are consumed in the southern (39%) and western states (38%) [16]. This might be partially explained by the high concentration of people of Hispanic origin populating the southern geographic region of the US. For example, states with the largest population of Hispanics in 2020 included five of the eight southernmost states (Florida, Texas, Nevada, Arizona, and California), according to the US Department of Health and Human Services Office of Minority Health [19].

Compared with non-consumers, we observed that pregnant women consuming ≥1 times a week of dried beans had a higher HEI score that corresponded to increased intake of protein, fat (including monounsaturated and polyunsaturated fat), and dietary fiber (both soluble and insoluble fractions). For the latter, the current recommendation for dietary fiber intake during pregnancy (14 g/1000 kcal) ranges from 25–36 g/day, depending on the mother’s age and trimester [9]. Recent work suggests that a high percentage (~70%) of pregnant women do not meet current dietary fiber recommendations [20]. In our cohort, bean consumption was associated with a substantial increase in total dietary fiber consumption from ~17–18 g/day in non-consumers to 24.4, 28.2, and 27.5 g/day in those with the highest intake frequency of dried beans, chili, and bean soup, respectively. Fiber consumption during pregnancy may benefit both the mother and developing fetus through improved glycemic control [21], a lower risk of pre-eclampsia [22], and promotion of healthy gestational weight gain (GWG) [23]. In a previous randomized controlled trial that investigated intake of a high-fiber diet (>30 g/d) during pregnancy to prevent excess GWG, Hull et al. reported that increased fiber intake resulted in lower GWG and lower weight retention 1 year postpartum in participants who continued to eat high-fiber foods [24]. 

In our supplemental analysis, despite their moderately higher energy intake, groups with the highest consumption of chili and dried beans did not have significantly different BMI from other groups with lower consumption. However, the group consuming the greatest amount of bean soup had a significantly lower BMI compared to those who did not frequently consume bean soup. This finding was supported by an experimental study showing an inverse correlation between soup intake and BMI after adjusting for the total energy intake and other covariates, which could potentially be explained by the involvement of soup consumption with slower gastric emptying or increased glycemic response [25]. 

Additionally, bean intake was associated with lower consumption of added sugar, a nutrient of particular concern that has been related to adverse pregnancy outcomes and the long-term health of offspring [26]. It is estimated that 70% of pregnant women in the US exceed the current recommendations for dietary added sugar [9]. In a previous prebirth cohort of 103,119 women, Maslova et al. reported a strong association between added sugar consumption and self-reported GWG [27]. Finally, higher bean consumption was associated with increased intake of a range of micronutrients including iron, potassium, magnesium, and folate. Adequate folate intake is crucial during early pregnancy [28] to protect against fetal neural tube defects, as well as anemia and peripheral neuropathy in mothers [29]. Beans have a high folate content. According to a previous study that explored the nutrient content of different pulses, pinto beans had the highest concentration of folate per serving (147 µg/0.5 cup) among the bean category, followed by chickpeas (141 µg/0.5 cup), adzuki beans (140 µg/0.5 cup), black beans (128 µg/0.5 cup), and navy beans (127 µg/0.5 cup) [30].

Total fiber and insoluble fiber among chili consumers became insignificant when calories were adjusted for. Although total fiber intake increased with more chili intake, energy intake increased as well. Similarly, several micronutrients including iron, potassium, vitamin D, and magnesium become insignificant when energy intake was adjusted for among chili consumers. Adjusting micronutrients for energy intake is important as it allows for better analysis of the diet composition rather than only total micronutrient intake [31]. Without adjusting for energy intake, it may act as a confounding variable due to its strong association with nutrients [32]. This helps to prevent misleading associations between bean consumption and micronutrient intake.

### Strengths and Limitations

One strength of our study was the relatively large sample size. This allowed us to estimate more reliable mean values to analyze the variations of bean consumption among various groups. Another strength of our study was that we were able to separate different ways of bean consumption including dried beans, chili, and bean soup. In addition, the detailed questions about frequency and serving size of bean consumption allowed us to estimate the total amount, which could provide in-depth understanding on this topic. Lastly, the use of a comprehensive FFQ allowed us to calculate macronutrients, micronutrients, and HEI as nutritional outcomes.

Along with the strengths, our research also had some limitations. In our study sample, 85.5% were White, which was higher than the national average and could introduce selection bias. Additionally, 78.8%were college-educated women, which might limit our statistical power to assess the impact of SES on bean consumption. There was also a lack of detailed information on chili and bean soup, including preparation methods and other ingredients (e.g., meat) used. In addition, there might have been potential dietary recall bias as some participants might have difficulties in accurately reporting the frequency and/or the amount of bean consumption over the past month. 

## 5. Conclusions

We conclude that bean consumption is low during pregnancy among US women. Consumption of beans one or more times per week might improve maternal diet quality. Our novel findings need replication in other studies with more diverse socio-demographics. Creating new public health practices including maternal nutrition enhancement through increased consumption of beans can help to develop new interventions for improved health of mothers and children, leading to more cost-effective care. Future research is needed to assess the health outcomes of the mother and the child related to bean consumption.

## Figures and Tables

**Table 1:** Socio-demographic characteristics of participants in the analytic sample.

	Total Sample (*n* = 2961)		Pregnant Women (*n* = 1444)		Non-Pregnant/ Non-Postpartum Women (*n* = 1517) **		*p*-Value
Characteristics *	*n* (%)	Mean ± SD	*n* (%)	Mean ± SD	*n* (%)	Mean ± SD	
**Age, years**		28.8 ± 5.6		28.8 ± 5.6			
**% of federal poverty level**		287.2 ± 222.0		257.2 ± 188.8		315.1 ± 245.5	<0.001
**Race/ethnicity**							<0.001
Non-Hispanic White	2070 (85.5)		1163 (83.5)		907 (88.3)		
Non-Hispanic Black	83 (3.4)		67 (4.8)		16 (1.6)		
Hispanic	157 (6.5)		94 (6.8)		63 (6.1)		
Asian/Pacific Islander/Other	110 (4.6)		69 (5.0)		41 (4.0)		
**Highest education level**							
1–8 years grade school	4 (0.3)		4 (0.3)				
High school	272 (20.9)		272 (20.9)				
1–3 years college	521 (40.0)		521 (40.0)				
College graduate	385 (29.5)		385 (29.5)				
Postgraduate	121 (9.3)		121 (9.3)				
**Employment status**							
Unemployed	499 (35.7)		499 (35.7)				
Employed	899 (64.3)		899 (64.3)				
**Household size**							<0.001
1–2 people	904 (31.0)		362 (25.8)		542 (35.7)		
3 people	833 (28.5)		528 (37.6)		305 (20.1)		
4 people	677 (23.2)		283 (20.2)		394 (26.0)		
≥5 people	507 (17.4)		231 (16.5)		276 (18.2)		
**Annual household income level (USD)**							<0.001
<25,000	601 (20.6)		314 (22.4)		287 (18.9)		
25,000–<40,000	596 (20.4)		317 (22.6)		279 (18.4)		
40,000–<60,000	619 (21.2)		318 (22.6)		301 (19.8)		
≥60,000	1105 (37.8)		455 (32.4)		650 (42.9)		
**WIC recipient status**							<0.001
Non-recipient	1811 (62.0)		825 (58.8)		986 (65.0)		
Recipient	1110 (38.0)		579 (41.2)		531 (35.0)		
**Regions of residency**							0.002
New England	137 (4.7)		68 (4.8)		69 (4.5)		
Middle Atlantic	368 (12.6)		166 (11.8)		202 (13.3)		
East North Central	553 (18.9)		282 (20.1)		271 (17.9)		
West North Central	251 (8.6)		136 (9.7)		115 (7.6)		
South Atlantic	475 (16.3)		232 (16.5)		243 (16.0)		
East South Central	170 (5.8)		80 (5.7)		90 (5.9)		
West South Central	318 (10.9)		152 (10.8)		166 (10.9)		
Mountain	257 (8.8)		137 (9.8)		120 (7.9)		
Pacific	392 (13.4)		151 (10.8)		241 (15.9)		
**Smoking during pregnancy**							
No	1246 (89.3)		1246 (89.3)				
Yes	150 (10.7)		150 (10.7)				
**Vegetarian diet**							0.114
No	2828 (95.9)		1389 (96.5)		1439 (95.3)		
Yes	122 (4.1)		51 (3.5)		71 (4.7)		

SD: standard deviation; WIC: Special Supplemental Nutrition Program for Women, Infants, and Children. * For some characteristics, the sum of categories is less than the total due to missing data. ** Some characteristics were not available for non-pregnant/non-postpartum women.

**Table 2:** Comparison of bean consumption between pregnant and non-pregnant/non-postpartum women.

	Pregnant Women		Non-Pregnant/ Non-Postpartum Women		
	*n* (%)	Mean ± SD	*n* (%)	Mean ± SD	*p*-Value *
**Frequency of dried bean consumption**					0.533
Never	649 (45.0)		694 (45.8)		
1 time a month	226 (15.7)		260 (17.2)		
2–3 times a month	331 (22.9)		323 (21.3)		
1 or more times a week	237 (16.4)		239 (15.8)		
**Serving size of dried bean consumption**					0.585
Never	649 (45.0)		694 (45.8)		
<1/2 cup/serving	213 (14.8)		243 (16.0)		
1/2–1 cup/serving	497 (34.5)		504 (33.3)		
>1 cup/serving	82 (5.7)		75 (4.9)		
**Amount of dried beans consumption,** **cup/week**		0.31 ± 0.57		0.32 ± 0.70	0.506
Meeting national recommendation **					0.676
Did not meet (<1 1/2 cups/wk)	1345 (93.3)		1410 (93.0)		
Met (≥1 1/2 cups/wk)	96 (6.7)		106 (7.0)		
**Frequency of chili consumption**					0.008
Never	932 (64.7)		911 (60.2)		
1 time a month	322 (22.4)		397 (26.2)		
2–3 times a month	156 (10.8)		154 (10.2)		
1 or more times a week	30 (2.1)		51 (3.4)		
**Serving size of chili consumption**					0.020
Never	932 (64.7)		911 (60.2)		
<1/2 cup/serving	96 (6.7)		90 (5.9)		
1/2 to 1 3/4 cups/serving	334 (23.2)		407 (26.9)		
>1 3/4 cups/serving	78 (5.4)		105 (6.9)		
**Amount of chili consumption, cup/week**		0.37 ± 0.57		0.44 ± 0.60	0.002
**Frequency of bean soup consumption**					0.639
Never	1176 (81.8)		1213 (80.6)		
1 time a month	218 (15.2)		246 (16.3)		
2–3 times a month	6 (0.4)		10 (0.7)		
1 or more times a week	37 (2.6)		36 (2.4)		
**Serving size of bean soup consumption**					0.249
Never	445 (30.9)		483 (32.0)		
<1 cup/serving	109 (7.6)		141 (9.3)		
1–2 cups/serving	815 (56.6)		819 (54.3)		
>2 cups/serving	70 (4.9)		66 (4.4)		
**Amount of bean soup consumption,** **cup/week**		0.10 ± 0.36		0.11 ± 0.39	0.805

SD: standard deviation. * *p*-value from chi-square tests for categorical variables and from ANOVA tests for continuous variables. ** Dietary Guidelines for Americans 2020–2025, based on a 2000-kcal diet.

**Table 3:** Maternal bean consumption during pregnancy by socio-demographics.

	Dried Bean				Chili				Bean Soup			
Characteristics	*n*	Mean ± SD (Cups/Week)	Overall *p*-Value	Pairwise Comparisons (LSD) *	*n*	Mean ± SD (Cups/Week)	Overall *p*-Value	PairwiseComparisons (LSD) *	*n*	Mean ± SD (Cups/Week)	Overall *p*-Value	Pairwise Comparisons (LSD) *
**Race/ethnicity**			<0.001				<0.001				0.849	
Non-Hispanic White	1160	0.28 ± 0.49		a	1159	0.14 ± 0.28		a	1160	0.10 ± 0.34		
Non-Hispanic Black	67	0.25 ± 0.38		a	67	0.33 ± 0.90		b	67	0.09 ± 0.41		
Hispanic	94	0.64 ± 1.06		b	94	0.15 ± 0.34		a	93	0.13 ± 0.36		
Asian/Pacific Islander/Other	69	0.37 ± 0.67		a	69	0.17 ± 0.31		a	69	0.11 ± 0.35		
**Education level**			0.086				0.011				0.210	
1–8 years grade school	4	0.12 ± 0.21		a, b	4	0.57 ± 0.41		a	4	0.05 ± 0.11		
High school	271	0.28 ± 0.61		a	271	0.18 ± 0.37		b	270	0.07 ± 0.25		
1–3 years college	519	0.28 ± 0.46		a	520	0.15 ± 0.37		b, c	519	0.08 ± 0.29		
College graduate	385	0.30 ± 0.51		a	385	0.11 ± 0.23		c	385	0.12 ± 0.45		
Postgraduate	121	0.43 ± 0.83		b	120	0.13 ± 0.32		b, c	121	0.12 ± 0.30		
**College education**			0.463				0.044				0.174	
Did not attend college	275	0.28 ± 0.60			275	0.18 ± 0.37			274	0.07 ± 0.25		
Attended college	1025	0.31 ± 0.54			1025	0.14 ± 0.32			1025	0.10 ± 0.36		
**Employment status**			0.464				0.339				0.601	
Unemployed	497	0.32 ± 0.62			498	0.17 ± 0.36			498	0.09 ± 0.29		
Employed	898	0.30 ± 0.55			896	0.15 ± 0.34			896	0.10 ± 0.38		
**Household size**			0.187				0.006				0.131	
1-2 people	361	0.32 ± 0.56		a, b	361	0.17 ± 0.43		a	362	0.13 ± 0.48		
3 people	527	0.28 ± 0.56		a	527	0.11 ± 0.23		b	525	0.09 ± 0.29		
4 people	283	0.29 ± 0.49		a, b	282	0.19 ± 0.40		a	283	0.07 ± 0.27		
≥5 people	230	0.38 ± 0.71		b	230	0.19 ± 0.34		a	229	0.13 ± 0.35		
**Annual household income level (USD)**			0.418				0.034				0.844	
<25,000	312	0.27 ± 0.55			313	0.20 ± 0.51		a	313	0.11 ± 0.33		
25,000–<40,000	317	0.35 ± 0.63			317	0.14 ± 0.23		b	314	0.11 ± 0.44		
40,000–<60,000	318	0.32 ± 0.59			318	0.15 ± 0.28		a, b	317	0.10 ± 0.31		
≥60,000	454	0.31 ± 0.54			452	0.13 ± 0.32		b	455	0.09 ± 0.34		
**WIC recipient status**			0.665				0.018				0.775	
Non-recipient	824	0.32 ± 0.57			822	0.14 ± 0.29			824	0.10 ± 0.36		
Recipient	577	0.30 ± 0.59			578	0.18 ± 0.41			575	0.10 ± 0.35		
**Regions of residency**			<0.001				0.037				0.600	
New England	68	0.26 ± 0.64		a, b, c	68	0.10 ± 0.24		a	68	0.13 ± 0.51		a, b
Middle Atlantic	166	0.31 ± 0.70		b, c	166	0.10 ± 0.26		a	165	0.14 ± 0.48		a
East North Central	281	0.22 ± 0.41		a, b	282	0.15 ± 0.26		a, b	281	0.07 ± 0.22		b
West North Central	136	0.15 ± 0.29		a	135	0.14 ± 0.31		a, b	136	0.07 ± 0.32		a, b
South Atlantic	232	0.36 ± 0.61		c	231	0.15 ± 0.52		a, b	231	0.10 ± 0.38		a, b
East South Central	79	0.43 ± 0.56		c	80	0.26 ± 0.36		c	80	0.10 ± 0.33		a, b
West South Central	152	0.42 ± 0.67		c	150	0.15 ± 0.27		a, b	152	0.10 ± 0.37		a, b
Mountain	137	0.32 ± 0.57		b, c	137	0.19 ± 0.36		b, c	136	0.11 ± 0.30		a, b
Pacific	150	0.38 ± 0.65		c	151	0.19 ± 0.35		b, c	150	0.13 ± 0.35		a, b
**Smoking during pregnancy**			0.122				0.623				0.344	
No	1243	0.32 ± 0.59			1242	0.15 ± 0.35			1242	0.10 ± 0.37		
Yes	150	0.24 ± 0.47			150	0.17 ± 0.35			149	0.08 ± 0.28		
**Vegetarian diet during pregnancy**			<0.001				0.230				<0.001	
No	1386	0.30 ± 0.56			1384	0.15 ± 0.36			1384	0.09 ± 0.35		
Yes	51	0.61 ± 0.83			51	0.22 ± 0.36			51	0.30 ± 0.52		

SD: standard deviation; WIC: Special Supplemental Nutrition Program for Women, Infants, and Children; LSD: Fisher’s least significant difference test method. * Labels a, b, c: for characteristics with 3 or more categories, bean consumption means sharing the same letters were not significantly different from each other. Bean consumption means having no letters in common were significantly different from each other.

**Table 4:** Maternal nutritional outcomes by the frequency of bean consumption during pregnancy.

	Frequency of Dried Beans Consumption					
	Never (a)	1 Time a Month (b)	2–3 Times a Month (c)	1 or More Times a Week (d)	Overall *p*-Value	Significant Pairwise Comparisons (LSD)
Nutritional Outcomes	Mean ± SD	Mean ± SD	Mean ± SD	Mean ± SD		
Total food energy, kcal/day	2125.7 ± 934.1	2053.3 ± 829.1	2136.7 ± 939.3	2384.8 ± 945.4	<0.001	a vs. d, b vs. d, c vs. d
Total carbohydrate, g/day	290.0 ± 145.5	278.1 ± 124.2	283.2 ± 136.1	316.3 ± 140.0	0.013	a vs. b
Energy from carbohydrate, %	54.2 ± 8.6	54.2 ± 8.4	52.9 ± 7.4	52.8 ± 6.9	0.028	a vs. c, a vs. d
Added sugar, tsp/day	21.0 ± 18.2	19.6 ± 17.5	19.6 ± 15.6	19.5 ± 16.2	0.495	
Added sugar, g/day	83.9 ± 72.9	78.2 ± 70.0	78.4 ± 62.3	78.1 ± 64.7	0.495	
Energy from added sugar, %	15.2 ± 8.7	14.9 ± 8.8	14.2 ± 7.2	12.6 ± 6.3	<0.001	a vs. d, b vs. d, c vs. d
Total fiber, g/day	17.4 ± 8.6	18.1 ± 8.8	18.9 ± 9.0	24.4 ± 10.3	<0.001	a vs. c, a vs. d, b vs. d, c vs. d
Total fiber adjusted for energy, g/1000 kcal	8.4 ± 2.6	9.0 ± 3.1	9.1 ± 2.6	10.5 ± 2.8	<0.001	a vs. b, a vs. c, a vs. d, b vs. d, c vs. d
Insoluble fiber, g/day	11.4 ± 5.9	11.9 ± 6.0	12.4 ± 6.2	16.1 ± 6.8	<0.001	a vs. c, a vs. d, b vs. d, c vs. d
Insoluble fiber adjusted for energy, g/1000 kcal	5.5 ± 1.9	5.9 ± 2.2	6.0 ± 1.9	7.0 ± 1.9	<0.001	a vs. b, a vs. c, a vs. d, b vs. d, c vs. d
Soluble fiber, g/day	5.8 ± 2.8	6.1 ± 2.9	6.3 ± 3.0	8.1 ± 3.5	<0.001	a vs. c, a vs. d, b vs. d, c vs. d
Soluble fiber adjusted for energy, g/1000 kcal	2.8 ± 0.8	3.0 ± 0.9	3.0 ± 0.8	3.5 ± 0.9	<0.001	a vs. b, a vs. c, a vs. d, b vs. d, c vs. d
Protein, g/day	79.9 ± 35.0	79.1 ± 34.8	83.3 ± 37.1	93.4 ± 36.7	<0.001	a vs. d, b vs. d, c vs. d
Energy from protein, %	15.4 ± 3.2	15.5 ± 3.3	15.8 ± 3.1	15.9 ± 2.6	0.047	a vs. c, a vs. d
Total fat, g/day	76.9 ± 37.7	74.4 ± 34.2	79.8 ± 38.4	89.3 ± 37.8	<0.001	a vs. d, b vs. d, c vs. d
Energy from total fat, %	32.6 ± 6.8	32.5 ± 6.6	33.5 ± 6.1	33.7 ± 5.5	0.036	a vs. c, a vs. d, b vs. d
Monounsaturated fat, g/day	27.9 ± 13.6	27.0 ± 12.4	29.2 ± 14.0	32.9 ± 13.9	<0.001	a vs. d, b vs. d, c vs. d
Energy from monounsaturated fat, %	11.9 ± 2.7	11.9 ± 2.7	12.3 ± 2.4	12.5 ± 2.3	0.002	a vs. c, a vs. d, b vs. c, b vs. d
Polyunsaturated fat, g/day	14.9 ± 7.9	14.5 ± 7.6	15.7 ± 8.3	18.5 ± 7.9	<0.001	a vs. d, b vs. d, c vs. d
Energy from polyunsaturated Fat, %	6.4 ± 2.0	6.4 ± 1.9	6.7 ± 1.8	7.1 ± 1.7	<0.001	a vs. c, a vs. d, b vs. d, c vs. d
Saturated fat, g	28.4 ± 15.2	27.3 ± 13.3	28.9 ± 15.0	31.2 ± 15.1	0.029	a vs. d, b vs. d
Energy from saturated fat, %	12.0 ± 3.0	11.9 ± 2.8	12.0 ± 2.7	11.6 ± 2.5	0.418	
Iron, mg/day	16.6 ± 7.6	16.4 ± 7.0	16.7 ± 7.2	19.6 ± 7.8	<0.001	a vs. d, b vs. d, c vs. d
Iron adjusted for energy, mg/1000 kcal	8.0 ± 2.3	8.2 ± 2.3	8.0 ± 1.9	8.4 ± 2.0	0.109	a vs. d, c vs. d
Potassium, mg/day	3402.6 ± 1719.2	3331.3 ± 1565.9	3410.6 ± 1565.1	3976.4 ± 1663.7	<0.001	a vs. d, b vs. d, c vs. d
Potassium adjusted for energy, mg/1000 kcal	1610.6 ± 380.1	1623.8 ± 393.1	1617.6 ± 340.3	1680.3 ± 310.7	0.084	a vs. d, c vs. d
Folate, mcg/day	249.9 ± 141.6	253.4 ± 133.6	266.6 ± 128.5	352.1 ± 166.3	<0.001	a vs. d, b vs. d, c vs. d
Folate adjusted for energy, mcg/1000 kcal	118.4 ± 39.0	124.0 ± 41.3	127.1 ± 34.1	149.7 ± 44.6	<0.001	a vs. d, b vs. d, c vs. d
Vitamin D, mcg/day	7.9 ± 6.6	7.5 ± 5.4	7.4 ± 5.2	7.6 ± 5.3	0.471	
Vitamin D adjusted for energy, mcg/1000 kcal	3.6 ± 2.1	3.6 ± 1.8	3.4 ± 1.6	3.1 ± 1.5	<0.001	a vs. d, b vs. d
Magnesium, mg/day	331.4 ± 161.5	325.4 ± 145.5	336.5 ± 149.5	398.6 ± 161.5	<0.001	a vs. d, a vs. c, b vs. d, c vs. d
Magnesium adjusted for energy, mg/1000 kcal	157.4 ± 34.1	159.4 ± 37.5	160.1 ± 31.6	169.3 ± 31.6	<0.001	a vs. d, b vs. d, c vs. d
HEI-2005 total core	63.6 ± 9.1	64.8 ± 9.5	65.4 ± 8.9	67.5 ± 7.8	<0.001	a vs. c, a vs. d, b vs. d, c vs. d
	**Frequency of chili consumption**					
	**Never (a)**	**1 time a month (b)**	**2–3 times a month (c)**	**1 or more times a week (d)**	**Overall *p*-value**	**Significant pairwise comparisons (LSD)**
**Nutritional outcomes**	**Mean ± SD**	**Mean ± SD**	**Mean ± SD**	**Mean ± SD**		
Total food energy, kcal/day	2071.3 ± 874.9	2264.1 ± 987.7	2341.5 ± 905.7	2899.5 ± 1322.7	<0.001	a vs. b, a vs. c, a vs. d, b vs. d, c vs. d
Total carbohydrate, g/day	279.3 ± 132.6	307.9 ± 149.6	310.1 ± 134.9	381.3 ± 204.2	<0.001	a vs. b, a vs. c, a vs. d, b vs. d, c vs. d
Energy from carbohydrate, %	53.8 ± 8.3	54.1 ± 7.7	52.8 ± 7.0	52.1 ± 8.5	0.284	
Added sugar, tsp/day	19.7 ± 16.9	21.6 ± 18.6	19.9 ± 15.8	24.2 ± 19.1	0.211	
Added sugar, g/day	78.8 ± 67.5	86.4 ± 74.5	79.6 ± 63.4	96.7 ± 76.3	0.211	
Energy from added sugar, %	14.8 ± 8.4	14.7 ± 8.0	13.3 ± 6.6	12.6 ± 6.5	0.115	a vs. c
Total fiber, g/day	18.1 ± 8.8	19.8 ± 9.9	21.1 ± 8.8	28.2 ± 13.4	<0.001	a vs. b, a vs. c, a vs. d, b vs. d, c vs. d
Total fiber adjusted for energy, g/1000 kcal	8.9 ± 2.8	8.9 ± 2.9	9.2 ± 2.7	10.1 ± 2.8	0.072	a vs. d, b vs. d
Insoluble fiber, g/day	11.9 ± 6.1	13.0 ± 6.7	13.8 ± 5.8	18.1 ± 8.9	<0.001	a vs. b, a vs. c, a vs. d, b vs. d, c vs. d
Insoluble fiber adjusted for energy, g/1000 kcal	5.9 ± 2.0	5.9 ± 2.0	6.1 ± 2.0	6.5 ± 1.9	0.293	
Soluble fiber, g/day	6.0 ± 2.9	6.6 ± 3.2	7.1 ± 3.0	9.8 ± 4.6	<0.001	a vs. b, a vs. c, a vs. d, b vs. d, c vs. d
Soluble fiber adjusted for energy, g/1000 kcal	3.0 ± 0.8	3.0 ± 0.9	3.1 ± 0.8	3.5 ± 1.0	0.002	a vs. d, b vs. d, c vs. d
Protein, g/day	79.3 ± 33.9	85.5 ± 37.9	92.0 ± 35.6	114.2 ± 56.6	<0.001	a vs. b, a vs. c, a vs. d, b vs. d, c vs. d
Energy from protein, %	15.6 ± 3.2	15.4 ± 3.1	15.8 ± 2.4	15.8 ± 3.4	0.581	
Total fat, g/day	75.9 ± 36.0	82.2 ± 40.4	87.2 ± 35.4	108.6 ± 48.0	<0.001	a vs. b, a vs. c, a vs. d, b vs. d, c vs. d
Energy from total fat, %	32.9 ± 6.7	32.7 ± 6.1	33.6 ± 5.8	34.2 ± 6.2	0.339	
Monounsaturated fat, g/day	27.7 ± 13.0	29.9 ± 14.7	32.0 ± 13.0	40.1 ± 18.0	<0.001	a vs. b, a vs. c, a vs. d, b vs. d, c vs. d
Energy from monounsaturated fat, %	12.0 ± 2.7	11.9 ± 2.4	12.4 ± 2.4	12.6 ± 2.6	0.205	
Polyunsaturated fat, g/day	15.0 ± 7.8	16.2 ± 8.6	17.3 ± 7.7	20.7 ± 8.8	<0.001	a vs. b, a vs. c, a vs. d, b vs. d, c vs. d
Energy from polyunsaturated Fat, %	6.5 ± 1.9	6.5 ± 1.9	6.7 ± 1.9	6.8 ± 2.0	0.505	
Saturated fat, g	27.6 ± 14.3	30.0 ± 16.0	31.4 ± 14.1	39.5 ± 19.0	<0.001	a vs. b, a vs. c, a vs. d, b vs. d, c vs. d
Energy from saturated fat, %	11.9 ± 2.9	11.8 ± 2.7	12.0 ± 2.5	12.2 ± 2.5	0.868	
Iron, mg/day	16.4 ± 7.2	17.6 ± 7.4	19.1 ± 8.0	23.4 ± 11.5	<0.001	a vs. b, a vs. c, a vs. d, b vs. c, b vs. d, c vs. d
Iron adjusted for energy, mg/1000 kcal	8.1 ± 2.1	8.0 ± 2.0	8.4 ± 2.4	8.3 ± 2.4	0.300	
Potassium, mg/day	3318.9 ± 1546.9	3678.4 ± 1816.7	3845.0 ± 1688.8	4931.1 ± 2206.2	<0.001	a vs. b, a vs. c, a vs. d, b vs. d, c vs. d
Potassium adjusted for energy, mg/1000 kcal	1616.9 ± 369.1	1635.5 ± 359.7	1639.8 ± 358.6	1726.8 ± 199.7	0.342	
Folate, mcg/day	254.9 ± 135.9	292.0 ± 164.9	302.0 ± 139.5	391.8 ± 180.7	<0.001	a vs. b, a vs. c, a vs. d, b vs. d, c vs. d
Folate adjusted for energy, mcg/1000 kcal	124.1 ± 39.1	130.2 ± 46.8	129.4 ± 36.2	141.0 ± 38.2	0.015	a vs. b, a vs. d
Vitamin D, mcg/day	7.2 ± 5.2	8.4 ± 7.5	8.5 ± 6.1	9.8 ± 6.1	0.001	a vs. b, a vs. c, a vs. d
Vitamin D adjusted for energy, mcg/1000 kcal	3.4 ± 1.8	3.6 ± 2.0	3.6 ± 1.7	3.3 ± 1.6	0.703	
Magnesium, mg/day	328.4 ± 147.6	357.2 ± 178.1	374.5 ± 150.4	472.6 ± 200.7	<0.001	a vs. b, a vs. c, a vs. d, b vs. d, c vs. d
Magnesium adjusted for energy, mg/1000 kcal	160.3 ± 34.8	159.0 ± 33.2	161.3 ± 31.3	167.4 ± 27.4	0.583	
HEI-2005 total core	64.7 ± 9.2	64.8 ± 8.9	65.5 ± 8.2	65.2 ± 9.0	0.813	
	**Frequency of bean soup consumption**					
	**Never (a)**	**1 time a month (b)**	**2 or more times a month (c)**		**Overall *p*-value**	**Significant pairwise comparisons (LSD)**
**Nutritional outcomes**	**Mean ± SD**	**Mean ± SD**	**Mean ± SD**			
Total food energy, kcal/day	2123.3 ± 910.2	2294.3 ± 975.6	2508.3 ± 1020.7		0.002	a vs. b, a vs. c
Total carbohydrate, g/day	286.3 ± 139.8	307.3 ± 138.5	338.0 ± 134.9		0.010	a vs. b, a vs. c
Energy from carbohydrate, %	53.7 ± 8.2	53.6 ± 7.5	54.4 ± 6.6		0.826	
Added sugar, tsp/day	20.5 ± 18.0	19.4 ± 13.2	16.8 ± 8.8		0.280	
Added sugar, g/day	82.0 ± 72.2	77.6 ± 52.8	67.1 ± 35.2		0.280	
Energy from added sugar, %	14.9 ± 8.4	13.2 ± 6.4	10.8 ± 4.1		<0.001	a vs. b, a vs. c
Total fiber, g/day	18.1 ± 9.0	22.2 ± 9.6	27.5 ± 10.1		<0.001	a vs. b, a vs. c, b vs. c
Total fiber adjusted for energy, g/1000 kcal	8.7 ± 2.7	10.0 ± 2.8	11.5 ± 3.3		<0.001	a vs. b, a vs. c, b vs. c
Insoluble fiber, g/day	11.9 ± 6.1	14.8 ± 6.5	18.1 ± 6.5		<0.001	a vs. b, a vs. c, b vs. c
Insoluble fiber adjusted for energy, g/1000 kcal	5.7 ± 1.9	6.6 ± 2.0	7.6 ± 2.3		<0.001	a vs. b, a vs. c, b vs. c
Soluble fiber, g/day	6.1 ± 3.0	7.3 ± 3.2	9.2 ± 3.7		<0.001	a vs. b, a vs. c, b vs. c
Soluble fiber adjusted for energy, g/1000 kcal	2.9 ± 0.8	3.3 ± 0.9	3.8 ± 1.0		<0.001	a vs. b, a vs. c, b vs. c
Protein, g/day	80.8 ± 34.8	90.4 ± 39.6	100.9 ± 42.0		<0.001	a vs. b, a vs. c
Energy from protein, %	15.5 ± 3.1	15.9 ± 2.9	16.1 ± 2.5		0.054	a vs. b
Total fat, g/day	78.0 ± 36.8	84.0 ± 40.1	90.7 ± 44.2		0.013	a vs. b, a vs. c
Energy from total fat, %	33.1 ± 6.5	32.8 ± 6.2	32.1 ± 5.5		0.563	
Monounsaturated fat, g/day	28.4 ± 13.4	30.7 ± 14.4	33.2 ± 16.6		0.009	a vs. b, a vs. c
Energy from monounsaturated fat, %	12.1 ± 2.6	12.0 ± 2.5	11.8 ± 2.3		0.725	
Polyunsaturated fat, g/day	15.2 ± 7.8	17.0 ± 8.6	19.4 ± 9.8		<0.001	a vs. b, a vs. c
Energy from polyunsaturated Fat, %	6.5 ± 1.9	6.7 ± 1.8	7.0 ± 1.8		0.134	
Saturated fat, g	28.5 ± 14.7	30.0 ± 15.6	31.1 ± 15.8		0.259	
Energy from saturated fat, %	12.0 ± 2.9	11.6 ± 2.5	10.9 ± 2.3		0.006	a vs. b, a vs. c
Iron, mg/day	16.5 ± 7.2	19.4 ± 8.6	21.7 ± 7.1		<0.001	a vs. b, a vs. c
Iron adjusted for energy, mg/1000 kcal	8.0 ± 2.1	8.7 ± 2.3	9.0 ± 1.9		<0.001	a vs. b, a vs. c
Potassium, mg/day	3382.6 ± 1605.0	3869.1 ± 1803.0	4495.9 ± 1987.7		<0.001	a vs. b, a vs. c, b vs. c
Potassium adjusted for energy, mg/1000 kcal	1607.4 ± 366.4	1691.5 ± 345.0	1794.7 ± 287.0		<0.001	a vs. b, a vs. c
Folate, mcg/day	260.2 ± 144.5	306.5 ± 136.8	385.0 ± 172.7		<0.001	a vs. b, a vs. c, b vs. c
Folate adjusted for energy, mcg/1000 kcal	123.4 ± 40.2	136.2 ± 37.3	157.3 ± 51.9		<0.001	a vs. b, a vs. c, b vs. c
Vitamin D, mcg/day	7.5 ± 5.7	8.3 ± 6.9	8.6 ± 7.7		0.164	
Vitamin D adjusted for energy, mcg/1000 kcal	3.5 ± 1.9	3.5 ± 1.9	3.1 ± 1.8		0.412	
Magnesium, mg/day	331.0 ± 150.8	387.5 ± 179.0	442.2 ± 177.1		<0.001	a vs. b, a vs. c, b vs. c
Magnesium adjusted for energy, mg/1000 kcal	157.7 ± 33.6	170.4 ± 33.0	179.7 ± 33.4		<0.001	a vs. b, a vs. c
HEI-2005 total core	64.1 ± 9.0	67.2 ± 8.2	70.6 ± 8.6		<0.001	a vs. b, a vs. c, b vs. c

SD: standard deviation; LSD: Fisher’s least significant difference test method. Group labels: group a—never consume dried beans; group b—consume dried beans 1 time a month; group c—consume dried beans 2–3 times a month; group d—consume dried beans 1 or more times a week. SD: standard deviation; LSD: Fisher’s least significant difference test method. Group labels: group a—never consume chili; group b—consume chili 1 time a month; group c—consume chili 2–3 times a month; group d—consume chili 1 or more times a week. SD: standard deviation; LSD: Fisher’s least significant difference test method. Group labels: group a—never consume bean soup; group b—consume bean soup 1 time a month; group c—consume bean soup 2 or more times a month.

**Table 5:** The correlations between bean consumption amount during pregnancy and nutritional outcomes.

	Dried Beans Amount, Cup/Week			Chili Amount, Cup/Week			Bean Soup Amount, Cup/Week		
Nutritional Outcomes	r *	*p*-Value	Coefficient of Determination (r^2^)	r *	*p*-Value	Coefficient of Determination (r^2^)	r *	*p*-Value	Coefficient of Determination (r^2^)
Total food energy, kcal/day	0.157	<0.001	0.025	0.213	<0.001	0.045	0.081	0.002	0.007
Total carbohydrate, g/day	0.126	<0.001	0.016	0.197	<0.001	0.039	0.070	0.008	0.005
Energy from carbohydrate, %	−0.038	0.152	0.001	−0.006	0.815	<0.001	0.009	0.742	<0.001
Added sugar, tsp/day	0.023	0.391	0.001	0.094	<0.001	0.009	−0.034	0.195	0.001
Added sugar, g/day	0.023	0.391	0.001	0.094	<0.001	0.009	−0.034	0.195	0.001
Energy from added sugar, %	−0.084	0.002	0.007	−0.030	0.253	0.001	−0.091	0.001	0.008
Total fiber, g/day	0.320	<0.001	0.103	0.222	<0.001	0.049	0.200	<0.001	0.040
Insoluble fiber, g/day	0.316	<0.001	0.100	0.199	<0.001	0.040	0.199	<0.001	0.040
Soluble fiber, g/day	0.310	<0.001	0.096	0.249	<0.001	0.062	0.189	<0.001	0.036
Protein, g/day	0.173	<0.001	0.030	0.216	<0.001	0.047	0.107	<0.001	0.011
Energy from protein, %	0.033	0.210	0.001	0.011	0.683	<0.001	0.042	0.115	0.002
Total fat, g/day	0.165	<0.001	0.027	0.177	<0.001	0.031	0.069	0.009	0.005
Energy from total fat, %	0.048	0.071	0.002	0.003	0.905	<0.001	−0.018	0.485	<0.001
Monounsaturated fat, g/day	0.178	<0.001	0.032	0.185	<0.001	0.034	0.071	0.007	0.005
Energy from monounsaturated fat, %	0.068	0.010	0.005	0.017	0.509	<0.001	−0.013	0.629	<0.001
Polyunsaturated fat, g/day	0.186	<0.001	0.034	0.149	<0.001	0.022	0.104	<0.001	0.011
Energy from polyunsaturated fat, %	0.094	<0.001	0.009	−0.002	0.935	<0.001	0.056	0.034	0.003
Saturated fat, g	0.122	<0.001	0.015	0.158	<0.001	0.025	0.040	0.129	0.002
Energy from saturated fat, %	−0.028	0.294	0.001	−0.014	0.608	<0.001	−0.071	0.007	0.005
Iron, mg/day	0.190	<0.001	0.036	0.205	<0.001	0.042	0.138	<0.001	0.019
Potassium, mg/day	0.161	<0.001	0.026	0.219	<0.001	0.048	0.120	<0.001	0.014
Folate, mcg/day	0.286	<0.001	0.082	0.211	<0.001	0.045	0.155	<0.001	0.024
Vitamin D, mcg/day	0.002	0.925	<0.001	0.113	<0.001	0.013	0.021	0.437	<0.001
Magnesium, mg/day	0.193	<0.001	0.037	0.197	<0.001	0.039	0.134	<0.001	0.018
HEI-2005 total score	0.117	<0.001	0.014	0.030	0.262	0.001	0.130	<0.001	0.017

* Pearson correlation coefficient.

## Data Availability

The inherent data, code book, and analytic code cannot be made available as they were provided by the CDC and cannot be shared without permission. Researchers who are interested in using the data can contact the CDC directly.

## Supplementary Materials

The following supporting information can be downloaded at: https://www.mdpi.com/article/10.3390/nu15092234/s1, Table S1: Maternal Body Mass Index by the Frequency of Bean Consumption During Pregnancy.
