# Supplementary material for: Maternal Bean Consumption during Pregnancy: Distribution and Nutritional Outcomes
Source: Nutrients. 2023 May 8;15(9):2234. doi: 10.3390/nu15092234 (PMC10181266; doi:10.3390/nu15092234)
Supplement: Supplementary file 1 [file nutrients-15-02234-s001.zip › nutrients-2316839-supplementary.pdf]

Supplemental Table S1. Maternal Body Mass Index by the Frequency of Bean Consumption During Pregnancy.

	Frequency of dried bean consumption				
	Never (a)	1 time a month (b)	2-3 times a month (c)	1 or more times a week (d)	P-value
	Mean ± SD /n (%)	Mean ± SD /n (%)	Mean ± SD /n (%)	Mean ± SD /n (%)	
**Body mass index, kg/m², mean ± SD***	26.7 ± 7.3	26.1 ± 6.4	26.1 ± 6.5	26.8 ± 6.5	0.430
**Body mass index categories, n (%)****					0.685
Underweight/normal weight	321 (51.4)	109 (51.2)	167 (52.5)	116 (51.1)	
Overweight	136 (21.8)	51 (23.9)	82 (25.8)	55 (24.2)	
Obese	168 (26.9)	53 (24.9)	69 (21.7)	56 (24.7)	

SD: standard deviation.
* Analysis of variance.
** Chi-square test.

	Frequency of chili consumption				
	Never (a)	1 time a month (b)	2-3 times a month (c)	1 or more times a week (d)	P-value
	Mean ± SD /n (%)	Mean ± SD /n (%)	Mean ± SD /n (%)	Mean ± SD /n (%)	
**Body mass index, kg/m², mean ± SD***	26.5 ± 6.7	26.3 ± 7.1	26.4 ± 7.2	27.7 ± 8.7	0.773
**Body mass index categories, n (%)****					0.762
Underweight/normal weight	459 (51.2)	166 (54.1)	76 (50.3)	10 (37.0)	
Overweight	209 (23.3)	70 (22.8)	37 (24.5)	8 (29.6)	
Obese	228 (25.4)	71 (23.1)	38 (25.2)	9 (33.3)	

SD: standard deviation.
* Analysis of variance.
** Chi-square test.

	Frequency of bean soup consumption			
	Never (a)	1 time a month (b)	2 or more times a month (c)	P-value
	Mean ± SD /n (%)	Mean ± SD /n (%)	Mean ± SD /n (%)	
**Body mass index, kg/m², mean ± SD***	26.7 ± 6.9	25.4 ± 6.6	24.8 ± 5.8	0.015
**Body mass index categories, n (%)****				0.017
Underweight/normal weight	560 (49.6)	125 (59.8)	27 (65.9)	
Overweight	270 (23.9)	45 (21.5)	8 (19.5)	
Obese	299 (26.5)	39 (18.7)	6 (14.6)	

SD: standard deviation.
* Analysis of variance.
** Chi-square test.
